# Complete Genome Sequence of Sphingomonas paucimobilis Strain Kira, Isolated from Human Neuroblastoma SH-SY5Y Cell Cultures Supplemented with Retinoic Acid

**DOI:** 10.1128/MRA.01156-20

**Published:** 2021-02-11

**Authors:** Kohei Nishimura, Mirai Ikarashi, Yuji Yasuda, Mayu Sato, Marta Cano Guerrero, Josephine Galipon, Kazuharu Arakawa

**Affiliations:** a Keio University, Institute for Advanced Sciences, Tokyo, Japan; b Keio University, Graduate School of Media and Governance, Tokyo, Japan; c Keio University, Faculty of Environment and Information Studies, Tokyo, Japan; d Tsuruoka Chuo Prefectural High School, Tsuruoka, Japan; e University of York, Faculty of Sciences, York, United Kingdom; Indiana University, Bloomington

## Abstract

Because of its small size, Gram-negative Sphingomonas paucimobilis can pose a risk of nosocomial infection. We report the complete circular genome sequence of *S. paucimobilis* strain Kira, which was isolated from retinoic acid-supplemented SH-SY5Y human cell cultures, to be 3,917,410 bp (G+C content, 65.7%; 3,672 protein-coding sequences), with two plasmids (79,575 bp and 44,333 bp).

## ANNOUNCEMENT

The *Sphingomonas* group is unique among Gram-negative bacteria for its absence of lipopolysaccharides, which are replaced by glycosphingolipids. Cells are rod shaped, chemoheterotrophic, nonfermenting, and strictly aerobic (1). Sphingomonas paucimobilis is characterized by slowly motile, yellow-colony-forming, 0.7- by 1.4-μm cells that can pass through 0.2-μm filters, causing nosocomial infections (2–4). Strain Kira was isolated from penicillin- and streptomycin-supplemented human neuroblastoma SH-SY5Y cell cultures with bacterial contamination that originated from a 5 mg/ml retinoic acid solution used to induce neuronal differentiation.

The contaminated culture medium was spread onto Dulbecco’s modified Eagle’s medium (DMEM) agar containing 10% fetal bovine serum (DMEM-FBS) and cultured in 5% CO_2_ at 37°C. A colony was transferred to liquid DMEM-FBS and grown overnight under the same conditions. This culture was spread onto Luria-Bertani (LB) agar. A single yellow colony was isolated as strain Kira, cultured by shaking in liquid LB medium at 37°C, and harvested at an optical density at 600 nm (OD_600_) of 0.463. Genomic DNA was extracted using a Genomic-tip 500/G (Qiagen) and washed in high salt (1 M NaCl, 2 mM EDTA) and low ethanol (25% to 66.7%) to remove polysaccharides. For Illumina sequencing, the DNA was sheared by a Covaris M220 ultrasonicator targeting 550 bp; a library was prepared using the KAPA HyperPlus kit (Kapa Biosystems) and sequenced by a NextSeq 500 system in 75-bp paired-end high-output mode. Nanopore sequencing was done with unsheared DNA with a rapid barcoding kit (SQK-RBK004) and R9.4.1 flow cell (FLO-MIN106) on a GridION X5 system (Oxford Nanopore Technologies).

A total of 121,546 Nanopore reads were filtered using NanoFilt v2.7.1 (-q 7 --headcrop 50) (5), resulting in 119,143 reads (*N*_50_, 20,682 bp; median quality score, 11.6). The genome was assembled using Canu v2.0 (6), and duplicated ends were identified by BLAST and manually deleted. The raw Illumina reads were mapped to the assembled Nanopore data using BWA-MEM v0.7.11 (7), and three rounds of Pilon v1.23 error correction were performed (8). This increased BUSCO v4.0.5 (9) completeness from 32.3% (fragmented, 50.8%; missing, 16.9%) to 98.4% (fragmented, 1.6%; missing, 0%). This finding was consistent with the score of other published *S. paucimobilis* strains. The genome was annotated and rotated using DFAST (10). Default parameters were used except where otherwise noted.

The genome of *S. paucimobilis* strain Kira consists of 3,917,410 bp, with a G+C content of 65.7%, harboring 3,672 protein-coding sequences, 9 rRNAs, 60 tRNAs, and 1 CRISPR sequence. The closest genome based on *k-*mer analysis by PATRIC Genome Finder (11) was *S. paucimobilis* strain LCT-SP1 (GenBank accession number GCA_001029575.1) (12). In addition, two novel plasmids were found, namely, pKira_1 (length, 79,575 bp; G+C content, 63.6%; number of protein-coding sequences, 98; *repA*, absent) and pKira_2 (length, 44,333 bp; G+C content, 66.5%; number of protein-coding sequences, 57; *repA*, present).

Consistent with the culture conditions, the genome harbors a class A β-lactamase (13) and four aminoglycoside resistance genes, conferring broad resistance to penicillins and aminoglycosides. It also lacks the *gidB* gene, which confers sensitivity to aminoglycosides (14). Furthermore, heavy metal (arsenic, copper, cobalt, and nickel) resistance genes were detected, as well as components of the conjugation system. Finally, a predicted relaxase and *oriT* sequence were found on pKira_2 by oriTfinder (15).

### Data availability.

The complete genome and plasmid sequences of *S. paucimobilis* strain Kira were deposited in the DDBJ (accession numbers AP023323, AP023324, and AP023325) and in the Sequence Read Archive (SRA) (BioProject number PRJNA646346). The strain was deposited at the National Institute of Technology and Evaluation (NITE), Biological Resource Center (NBRC), Japan, under the accession number NBRC 114974.
